# Which Factors Promote Interprofessional Competence in Placements on a Training Ward? New Findings

**DOI:** 10.1007/s40670-026-02704-9

**Published:** 2026-03-30

**Authors:** Michael Hirth, Jutta Hinrichs, Stephanie Schaefer, Mira Mette

**Affiliations:** 1 Department of Medicine II, Medical Faculty Mannheim, University Medical Centre Mannheim, Heidelberg University, Theodor-Kutzer-Ufer 1-3, 68167 Mannheim, Germany; 2 School of Physiotherapy, Academy of University Medical Centre Mannheim, Mannheim, Germany; 3 Coordination of practical training for nurses, University Medical Centre Mannheim, Mannheim, Germany; 4 Division for Study and Teaching Development, Medical Faculty Mannheim, Heidelberg University, Mannheim, Germany

**Keywords:** Interprofessional training ward, Interprofessional competence, Interprofessional education, Medical education

## Abstract

**Introduction:**

Interprofessional training wards (ITWs) are established educational formats to foster interprofessional (IP) competence in health professions education. While previous research has demonstrated their overall effectiveness, little is known about which specific factors within ITW placements drive final levels and gains of IP competence and whether these mechanisms differ across professions.

**Methods:**

In an explorative study, 151 participants (105 medical students, 23 trainee nurses, 23 trainee physiotherapists) completed their placement on the ITW. Their self-reported data were used for hierarchical multiple regression analyses to identify predictive factors of perceived final levels and gains of IP competence at the end of the placement.

**Results:**

For medical students and trainee nurses, the perceived quality of IP interaction during the placement was the strongest predictor of both final level and gain of IP competence (*p* < .001 and *p* < .01, respectively). Frequency of IP interaction contributed to learning gain (*p* < .05 and n.s., respectively) but explained less variance than interaction quality. Initial IP competence predicted final level of IP competence for medical students but not gain of IP competence. No significant predictors of IP competence development were identified for trainee physiotherapists.

**Conclusion:**

This study advances IP education research by shifting the focus from the effectiveness of ITWs to the mechanisms underlying IP competence development. High-quality IP collaboration emerges as a key driver of perceived IP competence development. The findings highlight the importance of intentionally designing and facilitating IP interaction and suggest that learning mechanisms may differ across professions.

## Introduction

In recent decades, advances in medical science and the increasing use of interprofessional (IP) health care teams have contributed substantially to improved patient outcomes, including higher quality of care, enhanced patient safety, improved care coordination, and greater patient satisfaction [1–6]. IP care has been shown to reduce medical errors, improve clinical decision-making and support more holistic, patient-centered care, particularly in complex clinical contexts [5–7]. 

At the same time, health care delivery has become increasingly complex, multifaceted, and specialized [1]. Alongside physicians and nurses, a growing number of health professionals, such as physiotherapists, pharmacists, social workers, occupational therapists and dietitians are now routinely involved in patient care [8]. While this diversity offers considerable potential, effective collaboration across professions requires specific competencies to integrate different perspectives, expertise and professional cultures in daily clinical practice [1, 8]. 

To address these challenges, IP education has gained increasing importance as a strategy to prepare health profession students for collaborative practice [9–11]. IP education aims to strengthen IP competencies that enable students to work effectively with other professions and with patients to improve health outcomes [5, 7, 10, 12]. By defining IP competencies as “knowledge, skills, attitudes, and values that together shape the judgments and behaviors that are essential for collaborative practice” [13], the established Canadian Interprofessional Health Collaborative (CIHC) Competency Framework for Advancing Collaboration demonstrates strong alignment with the widely recognized Interprofessional Education Collaborative (IPEC) framework [14]. CIHC articulates six interdependent competency domains - relationship-focused care/services, team communication, role clarification and negotiation, team functioning, team differences/disagreements processing and collaborative leadership - that together describe the knowledge, skills, attitudes, and values required for effective collaborative practice across health and social care [13]. Grounded in inclusion, equity, and system complexity, the framework emphasizes person-partnered, relationship-centered collaboration and is designed to be adaptable across diverse educational, practice, and service contexts.

A range of IP educational settings has been developed to foster IP competencies across health professions education [7, 9]. These range from classroom-based and simulated learning activities [15] to practice-based formats embedded in real clinical care. Among these, practice-based IP education offers particularly authentic learning opportunities, as students from different professions collaborate directly in patient care within routine clinical settings [7, 16, 17]. IP training wards (ITWs) represent a prominent example of practice-based IP education [6, 18–20]. In these settings, students from multiple health professions jointly care for patients within a regular hospital environment while being supervised by trained facilitators [18]. ITWs offer authentic learning opportunities that support the development of IP communication, role clarity, shared responsibility, and teamwork [6, 7, 18, 19, 21]. However, the implementation of ITWs is resource-intensive, requiring substantial staffing, organizational support, and coordination [22, 23]. 

Although IP education and ITWs have been widely shown to improve IP competence, teamwork and patient-related outcomes [5–7], existing research has predominantly focused on demonstrating *whether* ITWs are effective. In contrast, there is limited empirical evidence on *which specific factors* within ITW placements are responsible for IP learning effects and how these mechanisms may differ across professions [24]. In particular, little is known about the relative importance of quantitative versus qualitative aspects of IP interaction, nor about potential differences between factors predicting final level of IP competence and those driving IP competence development during the placement.

To address this gap, the present study analyzes determinants of IP competence development during ITW placements, differentiating between students’ individual background factors and modifiable features of the learning environment. By shifting the focus from overall effectiveness to underlying learning mechanisms, this study aims to provide evidence-informed guidance for the design, facilitation, and optimization of ITWs.

## Material & Methods

### Study Design

After approval by the Ethics Committee II of the Medical Faculty Mannheim, Heidelberg University, students completing their placement on Mannheim’s ITW were invited to participate in this study. Immediately after completing their placement, students were asked to complete an anonymous paper-based questionnaire. The questionnaire included items assessing the participant’s background, their experience during the placement, interest in IP learning and collaboration, levels of knowledge about other professions and professional and IP competence (see Table 1 for details). Accordingly, all parameters were based on students’ self-assessments. A retrospective pretest/posttest design was applied. In line with this design, self-reported data were collected once after the end of the placement. Participants rated their current competence level and experiences (posttest) and were additionally asked to retrospectively assess how they would have rated themselves at the beginning of the placement (retrospective pretest). Students completed all questionnaire items (Table 1) for both the posttest and the retrospective pretest. In the context of this study, the retrospective pretest/posttest design was chosen to reduce response-shift bias by ensuring that participants evaluated their pre- and post-placement competence using the same internal reference frame, thereby increasing the sensitivity to perceived changes in IP competence [25–27]. 

**Table 1:** Overview of the questionnaire structure and variables

Questionnaire/item	Variable	Item types	Dependent variable?	Independent variable?
Modified ICCAS [29, 30]	IP competence^1^	Likert scale	• Final level of IP competence • Gain of IP competence	• IP competence before the placement
My ability to develop diagnostic and therapeutic measures was/is very good.	Professional competence^2,3^	Likert scale	No	Yes
My practical skills were/are very good.				
My knowledge of the areas of responsibility of my health profession (doctor, nurse or physiotherapist) and the various requirements my profession was/is very good.				
Do you work or have you worked in patient care as your main job, in a secondary position, or on a voluntary basis?	Extracurricluar work experience	Yes/ No	No	Yes
My knowledge of the areas of responsibility of profession 1 (doctor, nurse, or physiotherapist) and the different requirements for this profession was/is very good.	Knowledge about other professions^2,4^	Likert scale	No	Yes
My knowledge of the areas of responsibility of profession 2 (doctor, nurse, or physiotherapist) and the different requirements for this profession was/is very good.				
My interest in learning and working with other professions was/is very high.	Interest in learning & collaboration	Likert scale	No	Yes
There was frequent contact with students of the other professions during the placement.	Frequency of IP interaction during the placement	Likert scale	No	Yes
I found the collaboration with other professions during my placement to be very good.	Quality of IP interaction during the placement	Likert scale	No	Yes

Notes. ^1^ The overall IP competence scale showed very good internal consistency (Cronbach’s alpha = 0.86). ^2^ The participant’s knowledge about his or her own profession was included in the factor “professional competence” while the factor „knowledge about other professions” comprises only the knowledge about the other two professions depending of the participant’s own profession; ^3^ Cronbach’s alpha = 0.70 (mean inter-item correlation = 0.44); ^4^ Cronbach’s alpha = 0.50–0.59 (*r* = 0.33–0.41) depending on profession

The inclusion criteria were completion of Mannheim’s ITW placement and a signed informed consent. Overall, the five steps of the modified Knowledge Discovery in Databases (KDD) framework [28] were followed: (1) selection (creating a target data set including only ITW participants), (2) pre-processing (conducting descriptive analyses and excluding outliers), (3) transformation (reorganizing the data), (4) data analysis, (5) interpretation/evaluation of the results.

## Participants and Mannheim’s ITW

The ITW at the University Medical Centre Mannheim (Germany) was established in 2017 within the Department of Gastroenterology and Infectious Diseases as part of an IP curriculum [19, 29]. The ward comprises twelve inpatient beds and is organized as an authentic clinical learning environment in which IP teams, consisting of medical students, trainee nurses, and trainee physiotherapists, assume shared responsibility for comprehensive patient care.

Students work in IP teams and provide day-to-day patient care under the supervision of experienced, specially trained facilitators from each profession, who deliberately remain in the background to promote student autonomy while ensuring patient safety. Supervision follows a facilitative rather than directive approach, encouraging students to negotiate roles, communicate across professional boundaries, and jointly plan and evaluate care.

The daily routine on the ITW includes structured and recurring time slots for IP exchange, such as IP morning briefings, joint ward rounds, handovers and dedicated reflection sessions. These formalized IP opportunities are complemented by informal interactions in a shared team workspace, fostering continuous communication and collaboration.

Prior to the ITW placement, students are extensively prepared predominantly within their profession-specific education programs. Consequently, the mandatory ITW placements, varying in duration from one to six weeks depending on the profession, represent a central experiential learning opportunity for developing IP competence through real patient care.

## Measurement of Outcomes (Dependent Variables)

**Fig. 1:**
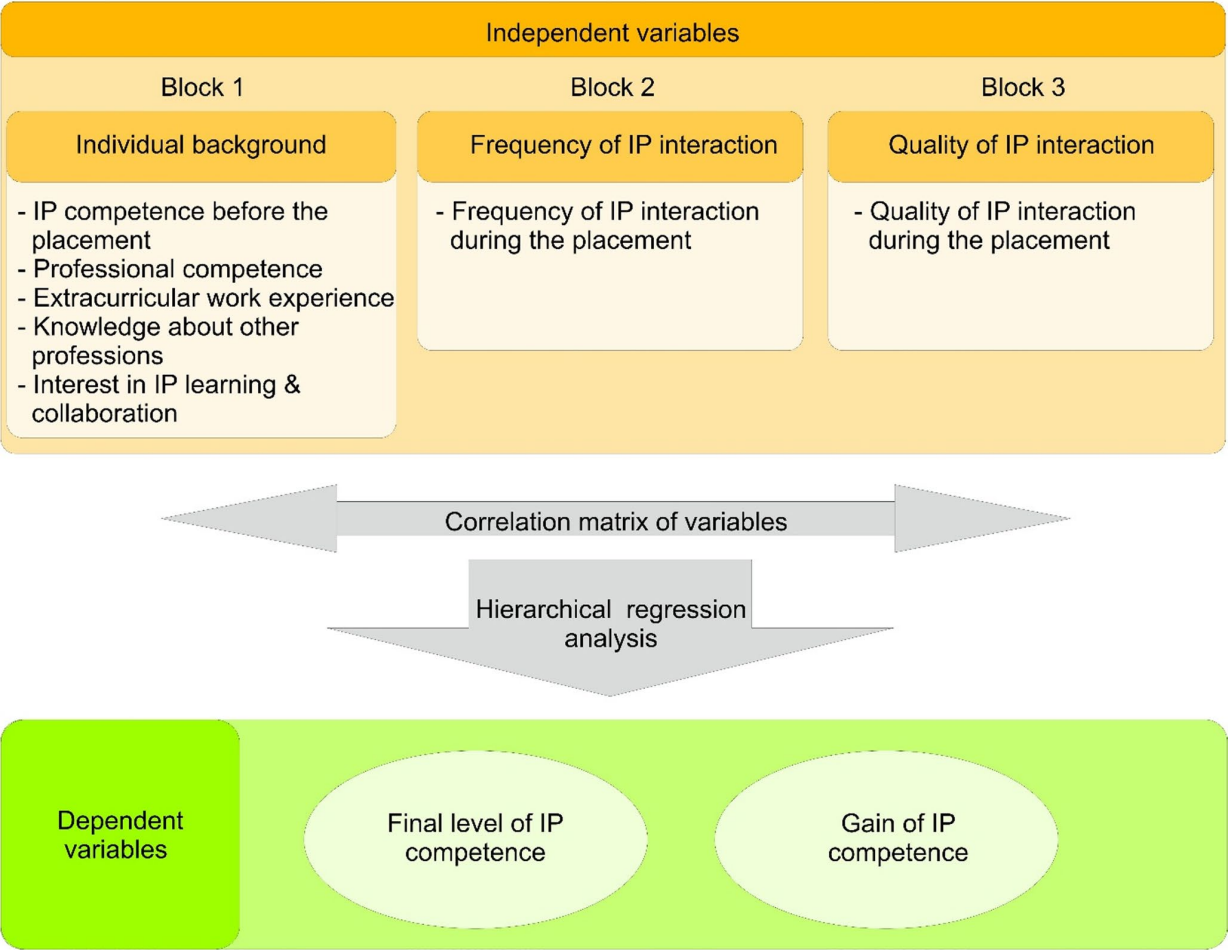
Overview of the study protocol

To examine which aspects of the ITW most strongly promote the development of IP competence, two dependent variables were defined: “final level of IP competence” and “gain of IP competence” (Fig. 1).

IP competence was assessed using the modified Interprofessional Collaborative Competency Attainment Survey (ICCAS) which is based on the CIHC framework [13, 29, 30]. The modified ICCAS was included in the questionnaire completed by students at the end of their ITW placement (Table 1).

Final level of IP competence was operationalized as the ICCAS_posttest_ score, measured on a Likert-type scale.

Gain of IP competence was calculated using a normalized gain score [31], reflecting the extent of competence development during the placement and allowing comparisons of learning gains across participants despite different baseline competence levels:

$$\:\mathrm{Ga}\mathrm{i}\mathrm{n}\:\mathrm{o}\mathrm{f}\:\mathrm{I}\mathrm{P}\:\mathrm{c}\mathrm{o}\mathrm{m}\mathrm{p}\mathrm{e}\mathrm{t}\mathrm{e}\mathrm{n}\mathrm{c}\mathrm{e}=\frac{{\mathrm{ICCAS}}_{\mathrm{posttest}}-{\mathrm{ICCAS}}_{\mathrm{retrospective}\text{}\mathrm{pretest}}}{{\mathrm{ICCAS}}_{\mathrm{scale}{\:maximum}}-{\mathrm{ICCAS}}_{\mathrm{retrospective}\text{}\mathrm{pretest}}}$$

## Measurement of Potential Predictors (Independent Variables)

Based on evidence from the literature [7, 10, 18] and our own empirical observations, a set of potential predictive factors (independent variables) that may influence IP competence development were identified, as shown in Fig. 1 and reported in detail in Table 1. Independent variables included IP competence before the placement, professional competence, knowledge about other professions, interest in IP learning and collaboration, and the perceived frequency and quality of IP interaction during the placement quantified by Likert-type scales. Extracurricular work experience (in patient care) was assessed as a dichotomous variable (yes/no).

IP competence before the placement was operationalized as the ICCAS_retrospective pretest_ score, reflecting participants’ self-assessed competence prior to the ITW placement. Professional competence was calculated as the mean score of items assessing practical skills, clinical reasoning and knowledge related to the participant’s own profession. Knowledge about other professions was computed as the mean score of items assessing knowledge about the two other professional groups represented on the ITW. Interest in IP learning and collaboration as well as the quality and frequency of IP interactions during the placement were derived from corresponding Likert-scale questionnaire items.

These independent variables were assigned to three analytical blocks (blocks 1–3), as illustrated in Fig. 1. They were grouped according to their chronological occurrence as “before ITW placement” (block 1; individual background) and “during ITW placement” (blocks 2 and 3). Factors occurring during the ITW were further differentiated into quantitative aspects (block 2) and qualitative aspects (block 3).

### Data Analysis

In order to analyze which independent variables are relevant predictors for the two dependent variables “final level of IP competence” and/or “gain of IP competence”, a multi-step approach was performed. First, a correlation matrix of the variables was created to identify potential predictors and issues that could affect the regression analyses. The strength of the associations was interpreted as weak (│r│ < 0.3), moderate (0.4 ≤ │r│ < 0.60) or strong (│r│ ≥ 0.6).

Further analyses were done separately for each profession due to huge differences in the number of participants in the groups. Hierarchical multiple regression analyses adding the three blocks of variables sequentially were used to assess their individual and incremental impact on the dependent variables. To determine the relevance of a statistically significant independent variable, i.e. how much it contributes to the total variance in the dependent variable when included in the model, the respective squared semipartial correlation (*∆R*^*2*^*)* was calculated. The values indicate the proportion of the total variance that is explained by this factor only. SPSS^®^, version 29 was used for statistical calculations with a significance level of *p* < 0.05.

## Results

Complete data were available from 151 participants across the three professions who completed their placement on Mannheim’s ITW (Table 2).

**Table 2:** Demographic characteristics of the survey participants

Profession	Medicine	Nursing	Physiotherapy
Number of participants	105	23	23
Female	53	17	15
Male	52	6	8

Table 3 presents the descriptive statistics and the correlation matrix for the two dependent variables and the independent variables considered to predict final level of IP competence and/or gain of IP competence during the placement. The direction and strength of the relationships between variables varied considerably, ranging from weak to strong, and differed across professions. For example, the association between gain of IP competence and perceived quality of IP collaboration was strong among trainee nurses (*r* = 0.60), moderate among medical students (*r* = 0.43) and weak for trainee physiotherapists (*r* = 0.24). Given these profession-specific differences, none of the independent variables were excluded from the subsequent regression analyses.

**Table 3:** Correlation matrix of the variables

					*Independent variables*							
DV/IV^1^	Profession	*Median*	*SD*	DV1	IV1	IV2	IV3	IV4	IV5	IV6	IV7	DV2
DV1: final level of IP competence^2^	medicine	4.37	0.40	1	0.52	0.28	0.09	0.08	0.24	0.34	0.54	x
	nursing	4.52	0.48	1	0.18	-0.01	-0.26	-0.10	0.39	0.57	0.81	x
	physio	4.49	0.35	1	0.30	0.29	0.10	0.10	0.16	0.33	0.35	x
IV1: IP competence before the placement^2^	medicine	3.75	0.53	0.52	1	0.37	0.03	0.46	0.38	0.03	0.20	-0.03
	nursing	3.70	0.65	0.18	1	0.29	-0.06	0.48	0.57	0.30	0.22	-0.28
	physio	3.35	0.85	0.30	1	0.67	-0.35	0.63	0.69	0.15	-0.03	-0.46
IV2: professional competence^2^	medicine	3.27	0.62	0.28	0.37	1	-0.18	0.23	0.16	0.14	0.14	-0.11
	nursing	4.15	0.46	-0.01	0.29	1	0.27	0.59	0.29	-0.18	-0.22	-0.22
	physio	4.07	0.41	0.29	0.67	1	-0.14	0.50	0.24	-0.03	-0.04	-0.14
IV3: extracurricular work experience^3^	medicine	56 / 49		0.09	0.03	-0.18	1	0.03	-0.00	0.18	0.17	0.09
	nursing	12 / 11		-0.26	-0.06	0.27	1	0.12	-0.03	-0.26	-0.43	-0.30
	physio	6 / 17		0.10	-0.35	-0.14	1	-0.48	0.02	-0.28	0.36	0.48
IV4: knowledge about other professions^2^	medicine	3.27	0.74	0.08	0.46	0.23	0.03	1	0.09	0.03	-0.19	-0.17
	nursing	3.28	0.65	-0.10	0.48	0.59	0.12	1	0.32	-0.23	-0.19	-0.31
	physio	3.28	0.81	0.10	0.63	0.50	-0.48	1	0.21	0.14	-0.43	-0.30
IV5: interest in IP learning & collaboration^2^	medicine	4.25	0.82	0.24	0.38	0.16	-0.00	0.09	1	0.31	0.37	-0.06
	nursing	4.13	1.22	0.39	0.57	0.29	-0.03	0.32	1	0.20	0.38	-0.07
	physio	3.87	1.01	0.16	0.69	0.24	0.02	0.21	1	-0.03	0.08	-0.42
IV6: frequency of IP interaction^2^	medicine	4.54	0.73	0.34	0.03	0.14	0.18	0.03	0.31	1	0.37	0.33
	nursing	4.61	0.58	0.57	0.30	-0.18	-0.26	-0.23	0.20	1	0.57	0.14
	physio	4.52	0.67	0.33	0.15	-0.03	-0.28	0.14	-0.03	1	-0.09	-0.06
IV7: quality of IP interaction^2^	medicine	4.66	0.53	0.54	0.20	0.14	0.17	-0.19	0.37	0.37	1	0.43
	nursing	4.26	1.01	0.81	0.22	-0.22	-0.43	-0.19	0.38	0.57	1	0.60
	physio	4.87	0.34	0.35	-0.03	-0.04	0.36	-0.43	0.08	-0.09	1	0.24
DV2: gain of IP competence	medicine	0.49	0.29	x	-0.03	-0.11	0.09	-0.17	-0.06	0.33	0.43	1
	nursing	0.58	0.40	x	-0.28	-0.22	-0.30	-0.31	-0.07	0.14	0.60	1
	physio	0.64	0.24	x	-0.46	-0.14	0.48	-0.30	-0.42	-0.06	0.24	1

*Notes.* Medicine = medical students (N = 105), nursing = trainee nurses (N = 23), physio = trainee physiotherapists (N = 23), SD = standard deviation; ^1^ DV = dependent variable / IV = independent variable; ^2^ range of scale between 1 (= complete disagreement) and 5 (= complete agreement), ^3^ dichotomous variable (yes/no). x = not analyzed as both variables are dependent variables

Hierarchical regression analyses were conducted separately for each profession to identify factors predicting the final level of IP competence (Tables 4 and 5) and gain of IP competence (Tables 4 and 6).

**Table 4:** Hierarchical regression analysis for “final level of IP competence” and “gain of IP competence” as dependent variables

		Final level of IP competence						Gain of IP competence					
	Predictor (block)	*F*	*p*	*R* ^*2*^	*∆F*	*p*	*∆R* ^*2*^	*F*	*p*	*R* ^*2*^	*∆F*	*p*	*∆R* ^*2*^
Medicine	individual background (1)	9.61	< 0.001 ***	0.33				1.53		0.07			
	frequency of IP interaction (2)	12.00	< 0.001 ***	0.42	16.47	< 0.001 ***	0.10	3.14	0.007 **	0.16	10.42	0.002 **	0.09
	Quality of IP interaction (3)	15.17	< 0.001***	0.52	20.13	< 0.001 ***	0.10	4.78	< .001 ***	0.26	12.49	< 0.001 ***	0.10
Nursing	individual background (1)	1.18	0.36	0.26				1.38	0.28	0.29			
	frequency of IP interaction (2)	2.19	0.099 **	0.45	5.63	0.031 *	0.19	1.12	0.39	0.30	0.17	0.69	0.01
	quality of IP interaction (3)	5.51	0.003 **	0.72	14.42	0.002 **	0.27	2.74	0.048 *	0.56	9.05	0.009 **	0.27
Physio	individual background (1)	0.73	0.61	0.18				2.45	0.08	0.42			
	frequency of IP interaction (2)	1.14	0.39	0.30	2.82	0.11	0.12	2.31	0.08	0.47	1.36	0.26	0.05
	quality of IP interaction (3)	1.40	0.28	0.40	2.39	0.14	0.10	2.02	0.12	0.49	0.59	0.45	0.02

*Notes.* Medicine = medical students, Nursing = trainee nurses, Physio = trainee physiotherapist; ∆R^2^ = change in R^2^ after entering a new block of predictors; *p < 0.05, **p < 0.01, ***p ≤ 0.001

**Table 5:** Regression results of the final model with the best fit and proportions of factors significantly contributing to predict “final level of IP competence”

	Profession	*B*	*SE*	*ß*	*t*	*p*	semi-partial *R*^*2*^
professional competence	medicine	0.03	0.05	0.05	0.59	0.56	
	nursing	0.18	0.19	0.17	0.07	0.35	
	physio^1^	0.15	0.28	0.18	0.56	0.59	
extracurricular work experience	medicine	-0.02	0.06	-0.02	-0.30	0.77	
	nursing	0.06	0.15	0.06	0.41	0.69	
	physio^1^	0.17	0.21	0.22	0.82	0.43	
knowledge about other professions	medicine	-0.05	0.05	-0.09	-1.03	0.31	
	nursing	0.03	0.15	0.04	0.18	0.86	
	physio^1^	0.06	0.14	0.14	0.43	0.67	
IP competence before the placement	medicine	0.39	0.07	0.53	5.70	< 0.001 ***	0.16
	nursing	-0.13	0.15	-0.17	-0.86	0.40	
	physio^1^	0.07	0.23	0.16	0.28	0.78	
interest in IP learning & collaboration	medicine	-0.09	0.04	-0.18	-2.12	0.04*	0.02
	nursing	0.04	0.07	0.10	0.51	0.62	
	physio^1^	-0.02	0.13	-0.05	-0.13	0.90	
frequency of IP interaction	medicine	0.13	0.04	0.24	2.93	0.004**	0.04
	nursing	0.19	0.15	0.26	0.18	0.22	
	physio^1^	0.21	0.11	0.39	1.81	0.09	
quality of IP interaction	medicine	0.29	0.06	0.39	4.49	< 0.001***	0.10
	nursing	0.35	0.09	0.75	3.80	0.002**	0.27
	physio^1^	0.39	0.25	0.38	1.55	0.14	

*Notes.* Medicine = medical students (*N* = 105), nursing = trainee nurses (*N* = 23), physio = trainee physiotherapists (*N* = 23); ^1^model = n.s. (values just for reference); *p < 0.05, **p < 0.01, ***p ≤ 0.001

**Table 6:** Regression results of the final model with the best fit with factors significantly contributing to predict “gain of IP competence”

	profession	B	SE	ß	t	*p*	semi-partial *R*^2^
IP competence before the placement	medicine	-0.03	0.06	-0.05	-0.45	0.65	
	nursing	-0.25	0.15	-0.41	-1.69	0.11	
	physio^1^	-0.10	0.15	-0.35	-0.66	0.52	
professional competence	medicine	0.04	0.05	0.09	0.92	0.36	
	nursing	0.06	0.19	0.06	0.29	0.78	
	physio^1^	0.09	0.18	0.15	0.49	0.63	
extracurricular work experience	medicine	0.00	0.05	0.00	0.01	0.99	
	nursing	-0.05	0.15	-0.07	-0.34	0.74	
	physio^1^	0.25	0.13	0.47	1.90	0.08	
knowledge about other professions	medicine	-0.04	0.04	-0.09	-0.85	0.40	
	nursing	-0.04	0.15	-0.07	-0.28	0.79	
	physio^1^	0.05	0.09	0.17	0.57	0.58	
interest in IP learning & collaboration	medicine	-0.05	0.04	-0.14	-1.33	0.19	
	nursing	0.02	0.08	0.06	0.27	0.79	
	physio^1^	-0.06	0.08	-0.27	-0.78	0.45	
frequency of IP interaction	medicine	0.09	0.04	0.22	2.23	0.03 *	0.04
	nursing	-0.13	0.16	-0.19	-0.83	0.42	
	physio^1^	0.09	0.07	0.24	1.18	0.26	
quality of IP interaction	medicine	0.21	0.06	0.38	3.53	< 0.001 ***	0.10
	nursing	0.29	0.10	0.74	3.01	0.009 **	0.27
	physio^1^	0.12	0.16	0.18	0.77	0.45	

*Notes.* Medicine = medical students (*N* = 105), nursing = trainee nurses (*N* = 23), physio = trainee physiotherapists (*N* = 23); ^1^model = n.s. (values just for reference); *p < 0.05, **p < 0.01, ***p ≤ 0.001

## Predictors of the Final Level of IP Competence

For medical students, the model including block 1 variables was significant, *F*(5, 99) = 9.61, *p* < 0.001, *R²* = 0.33. IP competence before the placement (*β* = 0.55, *p* < 0.001) and the knowledge about the other professions (*β* = −0.21, *p* = 0.03) were significant predictors of the final level of IP competence. Adding block 2 significantly improved model fit, *ΔF*(1,98) = 16.47, *p* < 0.001, *ΔR*^*2*^ = 0.10, with frequency of IP interaction emerging as an additional significant predictor (*β* = 0.34, *p* < 0.001). Inclusion of block 3 further improved the model, *ΔF*(1,97) = 20.13, *p* < 0.001, *ΔR*^*2*^ = 0.10, with the quality of IP interaction (*β* = 0.39, *p* < 0.001) and prior interest in IP learning and collaboration (*β* = -0.18, *p* = 0.04) emerging as additional significant predictors. The final model including blocks 1–3 accounted for 52% of the total variance in medical students’ final level of IP competence, *F*(7,97) = 15.17, *p* < 0.001 with the significant predictors IP competence before the placement, quality of IP interaction, frequency of IP interaction and interest in IP learning & collaboration explaining 16%, 10%, 4% and 2% of the total variance, respectively.

For trainee nurses, no significant predictor was revealed by using variables from block 1. By adding block 2, the model was significant, *F* [6, 16] = 2.19, *p* = 0.01, *R²* = 0.45, *ΔF* [1, 16] = 5.63, *p* = 0.03, *ΔR²* = 0.19, with frequency of IP interaction significantly predicting the final level of IP competence (*β* = 0.53, *p* = 0.03). The inclusion of block 3 significantly improved the final model, *F* [7, 15] = 5.51, *p* = 0.003, *ΔF* [1, 15] = 14.42, *p* = 0.002, *ΔR*^*2*^ = 0.27, which explained 72% of the total variance in trainee nurses’ final level of IP competence. Perceived quality of IP interaction acted as the only significant predictor (*β* = 0.75, *p* = 0.002) and accounted for 27% of the total variance.

No significant associations were found for the trainee physiotherapists.

## Predictors of the Gain of IP Competence

No significant predictors were identified in block 1 for any group. For medical students, block 2 significantly predicted gain of IP competence, *F*(6, 98) = 3.14, *p* = 0.007, *R²* = 0.16, *ΔF*(1, 98) = 10.42, *p* = 0.002, *ΔR²* = 0.09, driven by the inclusion of frequency of IP interaction (*β* = 0.33, *p* = 0.002). Adding block 3 further improved the final model, *ΔF*(1,97) = 12.45, *p* < 0.001, *ΔR*^*2*^ = 0.10. This final model accounted for 26% of the total variance in medical students’ gain of IP competence with quality of IP interaction (*β* = 0.38, *p* < 0.001) and frequency of IP interaction (*β* = 0.22, *p* = 0.03) as significant predictors, explaining 10% and 4% of the total variance.

For trainee nurses, only the final model including blocks 1–3 reached significance, *F* [7, 15] = 2.74, *p* = 0.048, *R²* = 0.56, *ΔF* [1, 15] = 9.05, *p* = 0.009. Quality of IP interaction was the sole significant predictor (*β* = 0.74, *p* = 0.009) explaining 27% of the total variance of trainee nurses’ gain of IP competence.

No significant predictors for gain of IP competence were identified for trainee physiotherapists.

## Discussion

IP education, and ITWs in particular, represent a well-established and increasingly studied educational format [16, 18–20, 32–35]. Nevertheless, there is still limited understanding of which specific factors within ITW placements drive IP learning and whether these mechanisms differ across professions [6, 18, 36]. The present study addresses this gap by systematically analyzing determinants of both, perceived final level and perceived gain of IP competence, differentiating between professions as well as between quantitative and qualitative aspects of IP interaction. Importantly, the outcome assessed in this study reflects students’ individual perceptions and self-assessments of their IP competence rather than externally validated or objectively measured IP competence. While self-perceptions do not necessarily correspond to actual performance, they represent an educationally meaningful construct, as they influence learners’ confidence, motivation, professional identity formation, and willingness to engage in IP collaboration [7, 37–39]. The following discussion therefore consistently refers to perceived IP competence and its development during the ITW placement.

Several key findings warrant closer discussion. First, and in line with previous research [6, 11, 36], an important predictor of perceived final level of IP competence was the self-reported level of IP competence before the ITW placement, particularly among medical students. This finding supports the assumption that prior exposure to IP learning and workplace situations facilitates later performance in IP settings [6, 11, 36]. At the same time, initial self-reported IP competence did not predict perceived gain of IP competence during the placement, suggesting that factors contributing to perceived final level of IP competence are not necessarily identical to those driving perceived competence development over time. This distinction between predictors of perceived level and gain of IP competence has rarely been made explicit in the literature, but is highly relevant from an educational perspective [5, 6, 29, 33–35, 40, 41]. 

Second, apart from students’ initial self-reported IP competence, both the frequency and, in particular, the quality of IP interaction during the placement were highly important for promoting IP competence. The quality of IP interaction was assessed based on students’ self-reports and therefore reflects perceived rather than objectively measured collaboration quality. Although trainees may not be able to judge all dimensions of interaction quality in an expert sense, these perceptions are educationally meaningful, as they shape engagement, motivation, and learning behavior [7, 37, 39]. Our findings suggest that IP interaction should be considered the core element of IP education and actively encouraged and facilitated [20]. The present study did not address how high-quality IP interactions can be fostered, as this lies beyond its scope. Nonetheless, evidence from the literature suggests that, alongside scheduled IP activities such as meetings and ward rounds, spontaneous and informal IP encounters are particularly valuable [42, 43]. Facilitators play a crucial role in helping students recognize the educational value of IP collaboration by enabling positive, content-rich interactions within a protected learning environment [44, 45]. Several studies highlighted that a safe learning environment with respectful interactions are key elements to stimulate high-quality IP communication and students’ development on a personal, professional and IP level [33, 46]. 

Third, although a certain level of profession-specific competence is clearly necessary to participate meaningfully in patient care on an ITW, the current data suggest that extensive mono-professional preparation alone is unlikely to substantially enhance perceived IP competence. At our institution, as likely at many others, prior to their placement on the ITW, students complete theoretical and semi-practical mono-professional training units aimed at consolidating profession-specific competencies [19]. However, the current findings indicate that this is unlikely to substantially promote perceived IP competence. In contrast, IP interactions on the ITW represent a key educational setting where the development of perceived IP competencies can be intentionally and substantially supported [6, 18, 20, 29]. 

Fourth, since we found difference predictors of perceived IP competence development between the professions, once may speculate, that the mechanisms underlying perceived IP competence development may differ between professions. While medical students and trainee nurses showed largely similar patterns, with perceived quality of IP interaction emerging as the most important predictor, no significant predictors were identified for trainee physiotherapists. This finding should be interpreted with caution due to the small number of trainee physiotherapists. However, it may also reflect differences in prior clinical experience and exposure to IP collaboration. In Germany, as well as in many other western countries, trainee physiotherapists’ regular clinical placements often already involve relatively autonomous patient care and regular IP contact, particularly when coordinating with physicians and nurses [47, 48]. Consequently, the ITW placement may offer fewer novel IP learning opportunities for this group, underscoring the need for profession-sensitive and context-specific approaches to IP education [19, 49]. 

These profession-specific patterns point to an aspect that has received comparatively little attention in the IP education literature but is highly relevant for educational design [5, 18, 20]. Differences in professional training programs and prior experiences with IP collaboration may shape how students engage in and benefit from IP learning environments [18, 20]. For example, assuming responsibility and actively speaking up within IP teams may initially be unfamiliar and challenging for students [29, 46, 50]. The ITW context, however, offers a unique opportunity to experience being heard, to strengthen professional confidence, and to appreciate the added value of IP collaboration for patient care [29, 34, 41, 46]. 

Several limitations of this study should be acknowledged. First, the single-center design limits the generalizability of the findings. Second, an important limitation concerns the exclusive reliance on self-report measures. The study therefore does not allow conclusions about objectively observable IP competence or actual collaborative performance. Self-assessments may be influenced by response styles, confidence levels, or social desirability. Future research should combine self-report instruments with observational or performance-based measures to better understand the relationship between perceived and demonstrated IP competence. Third, the relatively small and unequal sample sizes for trainee nurses and trainee physiotherapists reduce statistical power and increase the risk of type II errors. In particular, the absence of significant predictors for trainee physiotherapists should therefore be interpreted with caution. Forth, a further limitation relates to the variable ‘knowledge about other professions’, which demonstrated relatively low internal consistency (α = 0.50–0.59). Given the small number of items used to operationalize this construct, this finding should be interpreted cautiously.

## Conclusion

In conclusion, our findings reinforce the importance of IP interaction for perceived IP competence development on ITWs, while adding nuance by differentiating between perceived IP competence levels and gains, quantitative and qualitative aspects of interaction, and professions. Future research should further explore, with larger and more balanced samples, how such interactions can be reliably facilitated and how profession-specific learning mechanisms can be better addressed in IP education.
